# Temporal proteomic analyses of human lung cells distinguish high pathogenicity influenza viruses and coronaviruses from low pathogenicity viruses

**DOI:** 10.3389/fmicb.2022.994512

**Published:** 2022-10-10

**Authors:** Mahamud-ur Rashid, Kathleen K. M. Glover, Ying Lao, Victor Spicer, Kevin M. Coombs

**Affiliations:** ^1^Department of Medical Microbiology and Infectious Diseases, University of Manitoba, Winnipeg, MB, Canada; ^2^Manitoba Center for Proteomics and Systems Biology, Winnipeg, MB, Canada; ^3^Children’s Hospital Research Institute of Manitoba, John Buhler Research Center, Winnipeg, MB, Canada

**Keywords:** coronavirus, influenza virus, proteomics, TMT mass spectrometry, cell signaling, Rb-dependent E2F-mediated transcription

## Abstract

Newly re-emerging viruses are of significant global concern. In late 2019, a new coronavirus, SARS-CoV-2, emerged in China and soon spread worldwide, causing the COVID-19 pandemic, which to date has caused >6 M deaths. There has been a wealth of studies on this new virus since its emergence. The coronaviruses consist of many animal and human pathogens, with some of the human coronavirus, such as strain OC43, normally causing only mild cold-like symptoms. Viruses usurp host cellular processes to successfully replicate. We used tandem mass tag mass spectrometry-based proteomic analyses of human lung MRC-5 cells infected with OC43 for various periods of time to delineate virus-induced host cell alterations. Numerous proteins involved in lipid metabolism, molecular transport, small molecule biochemistry, cell death and survival, humoral immune response, and inflammatory response were dysregulated. Comparison of our findings to previous studies that examined a range of differentially pathogenic influenza A viruses (IAV), and to SARS-CoV-2 data, revealed that proteins involved in the cell cycle, cytokine signaling, DNA replication, and anti-inflammatory responses were generally similarly affected by virtually all tested IAV and CoV. However, proteins involved in necrosis, protein metabolism, ECM regulation, and signal transduction were generally different. In addition, the more pathogenic CoV and IAV activated Rb-dependent repression of E2F-mediated transcription, whereas less pathogenic influenza and coronaviruses either inhibited or had no effect on this pathway.

## Introduction

Viruses are obligate intracellular parasites that depend upon host processes for their replication. During their replication, viruses induce profound changes in the host cell’s protein repertoire (proteome). Viruses remain responsible for millions of deaths annually. In particular, the current COVID-19 pandemic, caused by the SARS-CoV-2 virus, has killed more than 6 million people since it emerged in Wuhan, China in late 2019. Rapid vaccine development and administration appear to have blunted the worst of this pandemic. Vaccines have been useful for ameliorating many infectious diseases, including smallpox, polio, and influenza (Delany et al., 2014, Nypaver et al., 2021). However, mutations that occur within viral and bacterial genomes as part of natural evolution can render vaccines ineffective. Thus, there is growing interest in understanding and exploiting cellular processes that viruses require that can be modulated to protect against these pathogenic organisms.

Some previous studies have identified some of the proteomic alterations induced by SARS-CoV-2, a BSL-3-level pathogen (Bojkova et al., 2020; Bouhaddou et al., 2020; Hekman et al., 2020; Stukalov et al., 2021). There are numerous other human coronaviruses (hCoV) that infect humans. To better understand host processes that differentiate between severe SARS-CoV-2 infection and infection by milder hCoV, we used tandem mass tag (TMT)-based mass spectrometry and determined proteomic alterations induced by OC43, a BSL-2 hCoV strain responsible for mild infections. OC43 infection induced dysregulation of several proteins involved in cell death and survival, humoral immune response, inflammatory response, lipid metabolism, molecular transport, and small molecule biochemistry in human lung MRC-5 cells. We also compared our OC43-induced proteomic signature to that induced by SARS1 and SARS-CoV-2 in A549 lung cells (Stukalov et al., 2021). Some dysregulated processes, such as autophagy, cytokine signaling, cell cycle, and anti-inflammatory response, were similarly affected by the mild hCoV and by the more pathogenic hCoV. However, several processes, such as apoptosis, antiviral response, cell cycle progression, and lipid and carbohydrate metabolism were differentially affected by OC43 and SARS-CoV-2.

## Materials and methods

### Cells and virus

Human lung MRC-5 cells (ATCC® #CCL-171) were maintained in Eagle’s medium (EMEM; ATCC #30-2003) supplemented with 10% Fetal Bovine Serum (FBS; Invitrogen) at 37°C in 5% CO_2_. Cells were trypsinized and sub-cultured at 1:4–1:6 ratios three times a week and were used between passage # 4 and 12. Human coronavirus OC43 stocks were propagated and titrated in MRC-5 cells. Cells were washed 2× in DMEM lacking FBS to remove serum and infected with OC43 at a multiplicity of infection (MOI) of 0.001 plaque-forming units (PFU) per cell. After 1 h adsorption in a 33.5°C incubator with periodic rocking, infected cells were overlaid with DMEM supplemented with 1 × l-glutamine, 1 × non-essential amino acids, 1 × sodium pyruvate, 0.25% BSA, and 1 μg/ml trypsin. Cells were incubated at 33.5°C and ¾ of the media were replaced 2 days post-infection (2dpi). Incubation continued until the cells showed >50% cytopathic effect. Supernatants were harvested and clarified of cell debris by centrifugation at 1,000 *× g* for 15 min. In some cases, virus was concentrated by centrifuging infected culture supernatants at 45,000 × *g* for 2 h in a Beckman JA-25.50 rotor and resuspended in small volumes of PBS. Virus titers were determined by immuno-focus assay as described (Lambert et al., 2008). Briefly, 50 μl aliquots of 1:10 dilutions were added to MRC-5 monolayers in 48-well plate. After 1 h adsorption in a 33.5°C incubator with periodic rocking, infected cells were overlaid with 1% Avicel® in 1× Medium 199 (M199) supplemented with 0.25% BSA and 1 μg/ml trypsin. Overlaid cells were incubated at 33.5°C until 38–40 hours post-infection (hpi), washed 1× with PBS, and fixed in PBS + 3% formaldehyde. After fixation for >6 h, cells were lysed with 0.1% NP-40 in TBST, blocked overnight in TBST + either 0.3% BSA or 5% skim milk, and probed with mouse α-OC43 N protein antibody (Sigma-Aldrich #MAB9013). Immune complexes were detected with an HRP-conjugated α-mouse 2° antibody (Cell Signaling #7076) and TrueBlue® (KPL #5510-0030) peroxidase substrate.

### Infection

For proteomic analyses, MRC-5 cells were infected at an MOI of 3 PFU per cell to ensure >95% synchronous infection. Virus was allowed to adsorb for 2 h before EMEM supplemented with 5% FBS was added. Mock and time-matched OC43-infected cells were harvested at 12, 24, and 48 hpi, to represent early, middle, and late events in virus infection, and proteomic analyses were performed on cell lysates obtained from three separate biological replicates.

### Protein quantification

Individual time-matched mock-infected and OC43-infected cells were harvested using sterile scrappers. Cells were pelleted by centrifugation at 600 × *g* for 8 min and washed 3× with sterile 1× PBS. Washed cells were lysed with 4% SDS in 100 mM HEPES buffer pH 8.5. Cell lysates were centrifuged at 14,000 × *g* for 15 min at 11°C to remove insoluble cellular components. Total lysate protein concentrations were determined using a commercial Bradford total protein estimation method (Pierce Biotechnology, Rockford, IL, United States).

### Tandem mass tags mass spectrometry analyses

Quantified proteins from 12, 24, and 48 hpi samples were digested into peptides using the SP3 (single-pot solid-phase-enhanced sample preparation) procedure described by Sielaff (Sielaff et al., 2017). Briefly, proteins were trypsin digested for 14 h at 37°C. Peptides were eluted and immediately submitted for TMT MS analysis. TMT labeling was performed as specified by the manufacturer (Thermo Scientific), except that TMT labels were dissolved in DMSO. Equivalent amounts of labeled samples within each TMT set were mixed prior to 2D LC/MS/MS.

An Agilent 1100 series LC system with UV detector (214 nm) and 1 mm × 100 mm XTerra C18, 5 μm column (Waters, Ireland) was used for pH 10 first dimension reversed-phase separation. A gradient of 1.80% acetonitrile per minute (0.1–59.9% acetonitrile in 30 min) was delivered at a flow rate of 150 μl/min. Both eluents A (water) and B (1:9, water:acetonitrile) contained 20 mM ammonium formate at pH 10. Twenty 1-min fractions were collected and concatenated into 10 (#1 mixed with # 11, etc.) to provide optimal orthogonal separation. These fractions were lyophilized and resuspended in 0.1% formic acid for the second dimension analysis.

Individual 6-plex TMT labeling was performed at each time point such that each 6-plex TMT reaction consisted of the three replicates of mock and three replicates of infected. Analyses of TMT-labeled peptides were performed on an Orbitrap Q Exactive HF-X instrument (Thermo Fisher Scientific, Bremen, Germany). The sample was introduced using an Easy-nLC 1,000 system (Thermo Fisher Scientific) at 1 μg per injection. Mobile phase A was 0.1% (v/v) formic acid and mobile phase B was 0.1% (v/v) formic acid in 80% acetonitrile (LC–MS grade). Gradient separation of peptides was performed on a C18 [Luna C18(2), 3 μm particle size (Phenomenex, Torrance, CA, United States)] column packed in-house in Pico-Frit (100 μm × 30 cm) capillaries (New Objective, Woburn, MA, United States). Peptides were separated by the following gradient: 5% phase B over 2 min, 5–7% increase of phase B over 2 min, 7–25% over 60 min, 25–60% over 15 min, 60–90% over 1 min, with a final elution of 90% B for 10 min at a flow rate of 300 nl/min.

Data acquisition on the Orbitrap Q Exactive HF-X instrument was configured for data-dependent method using the full MS/DD − MS/MS setup in a positive mode. The instrument is calibrated weekly, and polysiloxane (m/z = 445.12003), an internal control, is monitored during each run and does not vary more than 1 ppm. Spray voltage was set to 1.85 kV, funnel RF level at 40, and heated capillary at 275°C. Survey scans covering the mass range of 350–1,500 m/z were acquired at a resolution of 120,000 (at m/z 200), with a maximum ion injection time of 60 milliseconds, and an automatic gain control (AGC) target value of 3e6. For MS2 scan triggering, up to 20 of the most abundant ions were selected for fragmentation at 32% normalized collision energy, with intensity threshold kept at 6.3e4. AGC target values for fragment spectra were set at 1e5, which were acquired at a resolution of 30,000, with a maximum ion injection time of 80 ms and an isolation width set at 1.2 m/z. Dynamic exclusion of previously-selected masses was enabled for 20 s, charge state filtering was limited to 2–6, peptide match was set to preferred, and isotope exclusion was on.

### Peptide and protein identification and quantification

A database of protein sequences for hCoV and human (UniProt 2016; containing 20,168 human protein entries) was used for peptide/protein identification. For each time point, every 1D LC–MS run in the 2D-LC–MS experiment was converted into an MGF file using the Proteome Discoverer bundled tool. These were then concatenated into a single MGF per time point. These three concatenated MFGs files were each searched against the database using X!Tandem (cyclone 2012.10.01.1).

Standard peptide identification settings were used: single missed cleavage tryptic peptides were permitted, with a parent and fragment mass tolerance of 10 ppm. A fixed post-translational modification of C + 57.021 was applied, and variable PTMs including N-terminal acetylation, deamidation, phosphorylation, and oxidation were permitted. Peptide assignment into source proteins was managed by X!Tandem.

Peptide level TMT6 reporter tag intensities were integrated across a window of ±3 mDa each and corrected for isotopic overlap between channels using the supplied batch-specific correction matrix. Protein quantitation required at least two unique peptides of expectation values log(e) ≤ −1.5 each, yielding highly confident protein assignments with false discovery rate (FDR) = 0.1%. The sum of peptide level TMT6 reporter tag intensities for each protein was converted into a log_2_ scale for simplified differential analysis. Spectra (in MGF format) and an overall log_2_ protein expression matrix are available at the University of California, San Diego’s MassIVE archive^1^ under the accession MSV000089582.

### Immunoblotting

The levels of dysregulation of several of the identified proteins were validated by Western blotting, essentially as previously described (Coombs et al., 2010). Briefly, 30–50 μg of cell lysates were resolved in 10 or 12% SDS-PAGE, transferred to 0.2 μm Immobilon membranes, blocked overnight in TBST +5% skim milk, and probed with mouse primary anti-coronavirus OC43 N protein (Sigma-Aldrich #MAB9013), anti-CLIC1 (Sigma #MABN46), anti-HSPA5 (Sigma #MABC675), or rabbit anti-β-actin (Cell Signaling # 4970), anti-FHAD1 (OriGene #AP51667PU-N), anti-HLA-A (Genetex #GTX114080), anti-PSMA2 (Cell Signaling #2455), and anti-ZSWIM8 (Abbexa #ABX126815). Immune complexes were detected with secondary HRP-conjugated anti-mouse (Cell Signaling #7076) or anti-rabbit (Cell Signaling #7074) antibodies, and visualized on a GE Amersham Imager 680.

### Statistical and bioinformatics analyses

Individual protein differences between non-infected and each time-matched infected sample were converted to fold-changes and *p* values were calculated using Student’s *T*-test for grouped data to determine the level of significance. *Z*-scores for each replicate across all time points were calculated to classify proteins which were not considered significant by *T*-test. A value of *p* of <0.05 was used to select significantly dysregulated proteins and *Z*-score values of ≥ +1.96σ and ≤ −1.96σ were considered valid criteria for up- and downregulation. We use the term “up-regulated” to indicate proteins whose quantities are greater in the infected samples than in the mock non-infected samples, and may include proteins whose synthesis is increased, or whose turnover and loss are decreased, and/or a combination of the two processes. Similarly, “down-regulation” indicates proteins whose synthesis is decreased, or whose turnover and loss are increased, and/or a combination of the two processes.

Time-point-specific datasets containing protein IDs, fold-changes compared to mock-infected cells, and *p* values were uploaded into and analyzed by Ingenuity Pathway Analysis (IPA) software. The IPA database was used to classify all significantly regulated proteins based on protein type and subcellular location. Graphic representation of the distribution of molecules in different cellular compartments was done by manually modifying cell graphic vector downloaded from the IPA pathway designer tool. Information regarding the top affected bio-functions, canonical pathways, upstream molecules, and interconnecting networks were exported by performing a core analysis in IPA.

## Results

### OC43 causes specific temporal changes in the cellular proteome

**Figure 1:**
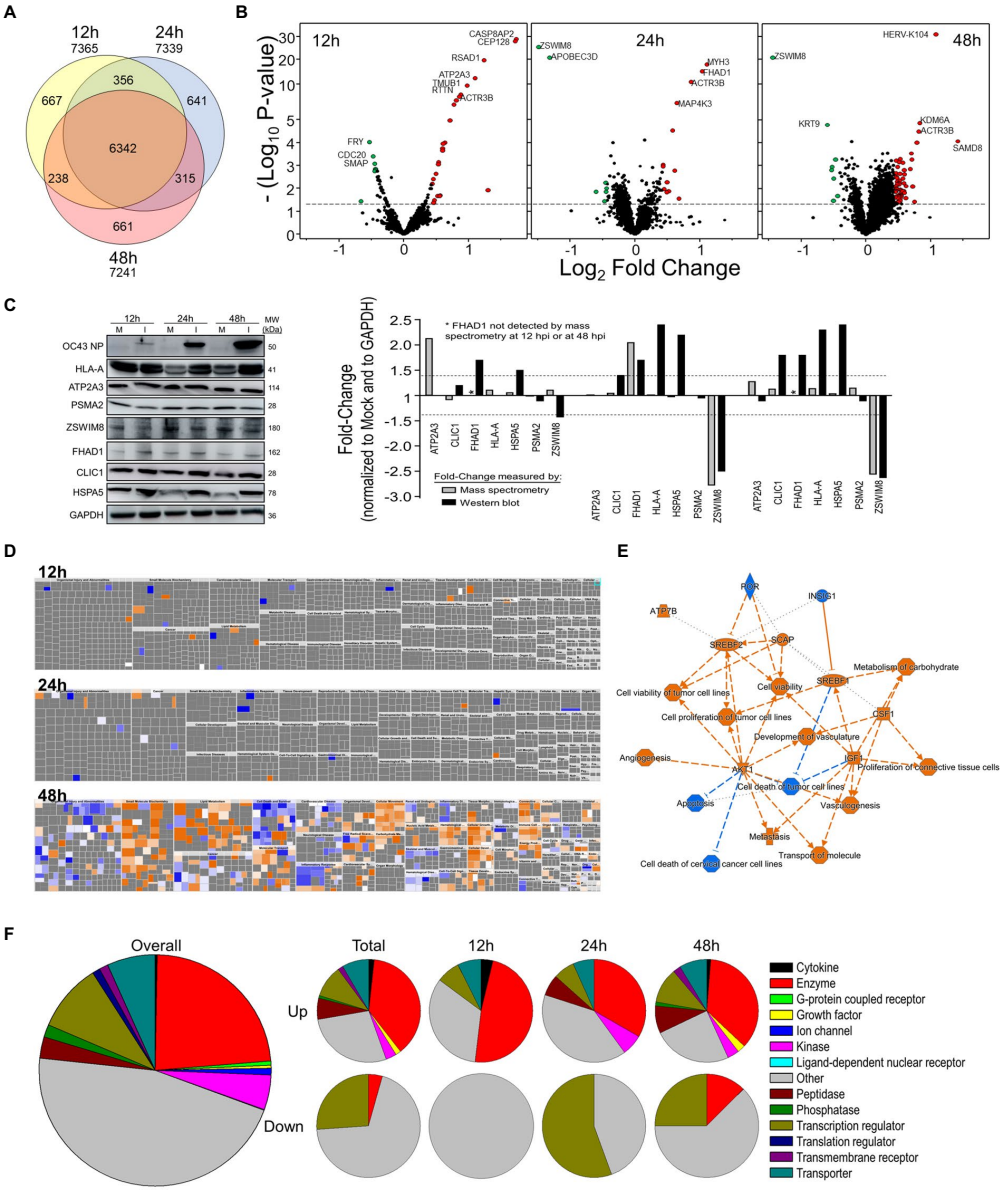
Tandem mass tag (TMT)-Mass spectrometry characterization of OC43-infected MRC-5 cells. **(A)** Venn diagram of numbers of proteins identified at each time point. **(B)** Volcano plots of dysregulated proteins. Horizontal dashed line indicates value of *p* of 0.05. **(C)** Western blot validation of protein dysregulation levels. The horizontal dashed lines represent ±1.4-fold-change cutoffs corresponding to Table 2. **(D)** Ingenuity Pathway Analysis (IPA)-predicted overall diseases and functions at each time point. **(E)** Major affected functions at 48 hours post-infection (hpi). **(F)** Gene ontologies of up- and downregulated processes.

We performed a non-biased 6-plex TMT mass spectrometry (MS)-based analysis of OC43-infected human lung MRC-5 cells, which led to the identification and measurement of 8,357 cellular proteins; 7,370 at 12 hpi, 7,344 at 24 hpi, and 7,246 at 48 hpi (Figure 1A). Of these, 1,510 proteins were considered significantly dysregulated at any of the three time points (Table 1). Many reported proteomic studies use protein cutoff values between ±1.25–2. As seen in Table 1, 317 proteins were significantly dysregulated >1.25-fold or < 0.8-fold (= 1/1.25), whereas only 11 proteins were significantly dysregulated >2-fold or < 0.5-fold (=1/2). Thus, we imposed a fold-change cutoff of ±1.33, which resulted in identification of 133 significantly dysregulated proteins, to enable meaningful bioinformatics analyses. The majority of proteins (27 at 12 hpi, 14 at 24 hpi, and 77 at 48 hpi) were upregulated. As indicated above, we use “up-regulated” to indicate proteins whose quantities are greater in the infected samples than in the mock non-infected samples, which may include proteins whose synthesis is increased, or whose turnover and loss are decreased, and/or a combination of the two processes. Similarly, “down-regulation” indicates proteins whose synthesis is decreased, or whose turnover and loss are increased, and/or a combination of the two processes. There were eight downregulated proteins at 12 hpi, nine at 24 hpi, and eight at 48 hpi (Table 1; Figure 1B). To validate the TMT-mass spectrometry results, we collected parallel mock- and OC43-infected cell lysates, resolved proteins by SDS-PAGE, and immunoprobed for several proteins (Figure 1C). Most of the highly dysregulated proteins were similarly regulated as measured both by Western blotting and by mass spectrometry. For example, FHAD1 was upregulated >1.7-fold at 24 hpi as measured by both methods and ZSWIM8 was downregulated >2.3-fold at both 24 and 48 hpi as measured by both methods. Many of the proteins were upregulated as measured by Western blot (i.e., HSPA5 at all time points, and CLIC1 and HLA-A at 24 and 48 hpi), but were not significantly dysregulated as measured by mass spectrometry, but this may relate to differences in techniques and what are measured (intact proteins by Western blot and tryptic fragments by mass spectrometry). Importantly, no proteins were significantly dysregulated in one direction by one method but significantly dysregulated in the opposite direction by the other method. Many more proteins and bio-functions were dysregulated at 48 hpi than at earlier time points (Figure 1D), and by 48 hpi these represented more than a dozen processes (Figure 1E). Enzymes were over-represented among the significantly upregulated proteins at all time points (Figure 1F). Cytokines were over-represented among the significantly upregulated proteins at 12 hpi, peptidases were over-represented among the significantly upregulated proteins at 24 and 48 hpi, and growth factors were over-represented among the significantly upregulated proteins at 48 hpi. Some of the most dysregulated proteins, with fold-change > ± 2, were CASP8AP2, CEP128, RSAD1, and ATP2A3 at 12 hpi, MYH3 and FHAD1 at 24 hpi, and SAMD8, HERV-K104, and ZSWIM8 at 48 hpi (Table 2). All of these except ZSWIM8 were upregulated. In addition, ACTR3 was upregulated >1.7-fold at all time points.

**Table 1:** Numbers of significantly dysregulated OC43-infected MRC-5 proteins.

	Total unique	12 hpi	24 hpi	48 hpi
Total proteins	8,357	7,370	7,344	7,246
Number significantly dysregulated				
and F.C. > 1.000	1,510	38	79	1,061
and F.C. < 1.000		37	236	155
and F.C. > 1.100	1,327	38	70	949
and F.C. < 0.90909		37	205	111
and F.C. > 1.250	317	36	30	219
and F.C. < 0.8000		22	19	15
and F.C. > 1.333	133	27	14	77
and F.C. < 0.750		8	9	8
**and F.C. > 1.400**	**77**	**21**	**10**	**42**
**and F.C. < 0.71429**		**3**	**3**	**6**
and F.C. > 1.500	36	15	6	12
and F.C. < 0.6667		1	3	2
and F.C. > 1.750	15	8	3	3
and F.C. < 0.5714		0	2	1
and F.C. > 2.000	11	5	2	2
and F.C. < 0.5000		0	2	1

Significance was determined by *T*-test and *Z*-score as detailed in Materials and Methods from three biological replicates. Bold indicates proteins identified in Table 2. hpi, hours post-infection.

**Table 2:** MRC-5 proteins dysregulated ≥1.4-fold by OC43 infection.

		12 h		24 h		48 h	
Gene	Protein	Fold-change	*p* value	Fold-change	*p* value	Fold-change	*p* value
CASP8AP2	CASP8-associated protein 2	**3.32**	2.3E−30				
CEP128	Centrosomal protein of 128 kDa	**3.27**	2.4E−29	1.02	0.815	1.09	0.075
COX7C	Cytochrome c oxidase subunit 7C, mitochondrial	**2.45**	0.012			1.03	0.704
RSAD1	Radical S-adenosyl methionine domain-containing protein 1, mitochondrial	**2.35**	3.7E−19			1.07	0.329
ATP2A3	Sarcoplasmic/endoplasmic reticulum calcium ATPase 3	**2.13**	2.3E−12	1.01	0.856	1.28	0.016
TMUB1	Transmembrane and ubiquitin-like domain-containing protein 1	**1.96**	3.9E−10	0.83	0.049	1.22	0.247
RTTN	Rotatin	**1.83**	3.7E−08				
ACTR3B	Actin-related protein 3B	**1.81**	9.8E−08	**1.82**	5.1E-11	**1.73**	3.2E-05
ALB	Serum albumin	**1.75**	3.6E−07	1.33	0.002	**1.48**	0.006
ZNF227	Zinc finger protein 227	**1.70**	1.3E−06				
RAG1	V(D)J recombination-activating protein 1	**1.63**	1.1E−05				
EXT1	Exostosin-1	**1.54**	1.0E−04	1.05	0.514	1.11	0.113
TBC1D8	TBC1 domain family member 8	**1.52**	1.1E−04				
ALKBH5	RNA demethylase ALKBH5	**1.51**	1.8E−04	1.10	0.194	1.06	0.423
DHX40	Probable ATP-dependent RNA helicase DHX40	**1.51**	2.3E−04				
SCD	Acyl-CoA desaturase	**1.47**	0.021	1.30	0.012	**1.61**	0.001
SQLE	Squalene monooxygenase	**1.46**	0.022	**1.41**	0.006	**1.60**	0.0003
BST1	Bone marrow stromal antigen 1	**1.44**	7.1E-04	1.11	0.267	1.13	0.021
ISOC2	Isochorismatase domain-containing protein 2	**1.44**	8.6E−04	1.07	0.293	1.25	0.054
ARFRP1	ADP-ribosylation factor-related protein 1	**1.43**	0.021	1.07	0.166	0.97	0.411
C17orf80	Uncharacterized protein C17orf80	**1.40**	0.002	1.10	0.235	1.25	0.083
MSMO1	Methylsterol monooxygenase 1	1.33	0.010	1.30	0.023	**1.41**	0.006
TMEM119	Transmembrane protein 119	1.24	0.049	1.04	0.604	**1.41**	0.001
RNF24	RING finger protein 24	1.21	0.049	**1.59**	0.030	**1.65**	0.038
CDC20	Cell division cycle protein 20 homolog	**0.71**	3.7E−04				
FRY	Protein furry homolog	**0.69**	9.0E−05	0.98	0.703	1.15	0.180
CCDC146	Coiled-coil domain-containing protein 146	**0.63**	0.035	0.89	0.091	0.96	0.158
MYH3	Myosin-3			**2.15**	3.3E-17		
FHAD1	Forkhead-associated domain-containing protein 1			**2.05**	1.4E-14		
MAP4K3	Mitogen-activated protein kinase 3	1.02	0.833	**1.56**	9.2E-07	1.08	0.209
MX1	Interferon-induced GTP-binding protein Mx1			**1.53**	0.002		
THY1	Thy-1 membrane glycoprotein	1.48	0.200	**1.49**	3.1E-05	1.54	0.093
RNF130	E3 ubiquitin-protein ligase RNF130			**1.43**	0.014	1.33	0.011
DDAH2	N(G),N(G)-dimethylarginine dimethylaminohydrolase 2	1.09	0.355	1.27	0.029	**1.48**	0.001
FABP3	Fatty acid-binding protein, heart	0.96	0.637	1.27	0.006	**1.52**	0.008
SQSTM1	Sequestosome-1	1.19	0.080	1.22	0.008	**1.42**	0.001
FASN	Fatty acid synthase	0.98	0.805	1.19	0.041	**1.42**	0.011
ARL2BP	ADP-ribosylation factor-like protein 2-binding protein	0.98	0.881	1.04	0.033	**1.44**	0.020
TCEAL7	Transcription elongation factor A protein-like 7	0.90	0.124	0.74	0.007		
ZNF132	Zinc finger protein 132			0.73	0.014	**0.71**	0.034
DEF6	Differentially expressed in FDCP 6 homolog	0.75	0.241	**0.66**	0.014		
APOBEC3D	DNA dC- > dU-editing enzyme APOBEC-3D			**0.40**	1.4E-20		
ZSWIM8	Zinc finger SWIM domain-containing protein 8	1.11	0.300	**0.36**	5.0E-26	**0.39**	1.9E-20
SAMD8	Sterile alpha motif domain-containing 8	1.04	0.648			**2.63**	0.0001
HERV-K104	Endogenous retrovirus group K member 104 Rec protein					**2.08**	1.8E-09
KDM6A	Lysine-specific demethylase 6A	1.09	0.467	1.14	0.555	**1.75**	1.3E-05
TAF6L	TAF6-like RNA polymerase II p300/CBP-associated factor-associated factor 65 kDa subunit 6 l	1.25	0.138	1.44	0.179	**1.70**	9.8E-05
AKR1C1	Aldo-keto reductase family 1 member C1	1.05	0.431	1.02	0.803	**1.62**	0.008
SECTM1	Secreted and transmembrane protein 1					**1.58**	0.001
PDCD4	Programmed cell death protein 4			1.04	0.496	**1.51**	0.026
RFTN2	Raftlin-2			1.02	0.821	**1.49**	0.002
PSMB3	Proteasome subunit beta type-3	1.07	0.519	1.06	0.385	**1.49**	0.015
AGT	Angiotensinogen					**1.48**	0.012
PEX13	Peroxisomal membrane protein PEX13	1.03	0.709	1.03	0.601	**1.46**	0.004
CD320	CD320 antigen	1.18	0.134			**1.46**	0.001
NQO1	NAD(P)H dehydrogenase [quinone] 1	0.94	0.511	1.07	0.445	**1.46**	0.013
HBA1	Hemoglobin subunit alpha	1.18	0.257	0.97	0.835	**1.45**	0.049
SOD2	Superoxide dismutase [Mn], mitochondrial	1.31	0.114	1.10	0.355	**1.45**	0.013
AKR1B1	Aldose reductase	0.97	0.748	1.09	0.412	**1.44**	0.012
HSPB1	Heat shock protein beta-1	0.89	0.241	1.08	0.299	**1.43**	0.021
PKM	Pyruvate kinase PKM	1.00	1.000	1.16	0.151	**1.43**	0.022
UBE2O	(E3-independent) E2 ubiquitin-conjugating enzyme	1.02	0.873	1.04	0.612	**1.42**	0.017
KIAA0513	Uncharacterized protein KIAA0513	1.06	0.556			**1.42**	0.027
CYP1B1	Cytochrome P450 1B1	1.18	0.195	1.13	0.081	**1.42**	0.002
SAR1A	GTP-binding protein SAR1a	1.01	0.913	1.11	0.276	**1.42**	0.034
GSTM2	Glutathione S-transferase Mu 2	0.95	0.592	1.04	0.577	**1.41**	0.020
PIR	Pirin	0.99	0.923	1.17	0.081	**1.41**	0.020
NFATC4	Nuclear factor of activated T-cells, cytoplasmic 4	1.01	0.868	0.93	0.208	**1.41**	0.021
RPAP1	RNA polymerase II-associated protein 1	1.01	0.935	1.16	0.116	**1.41**	0.033
PLIN2	Perilipin-2	0.85	0.131	1.02	0.640	**1.41**	0.004
KIAA1467	Uncharacterized protein KIAA1467			1.02	0.649	**1.41**	0.023
LPIN1	Phosphatidate phosphatase LPIN1	1.11	0.343	1.11	0.083	**1.41**	0.006
TKT	Transketolase	0.92	0.436	1.07	0.408	**1.40**	0.018
TOP2A	DNA topoisomerase 2-alpha	1.06	0.653	0.91	0.354	**0.71**	0.004
PTX3	Pentraxin-related protein PTX3	1.11	0.286	0.99	0.859	**0.70**	0.001
INCENP	Inner centromere protein	1.03	0.800	0.93	0.278	**0.69**	0.002
KRT9	Keratin, type I cytoskeletal 9	0.81	0.184	1.13	0.594	**0.66**	1.6E-05

Values determined from three replicates. Values ranked in descending order and from 12 to 48 h. Bold indicates proteins significantly (*p* < 0.05) dysregulated ≥ 1.40-fold, or ≤ 0.714.

### Cellular networks affected by OC43 infection

**Figure 2:**
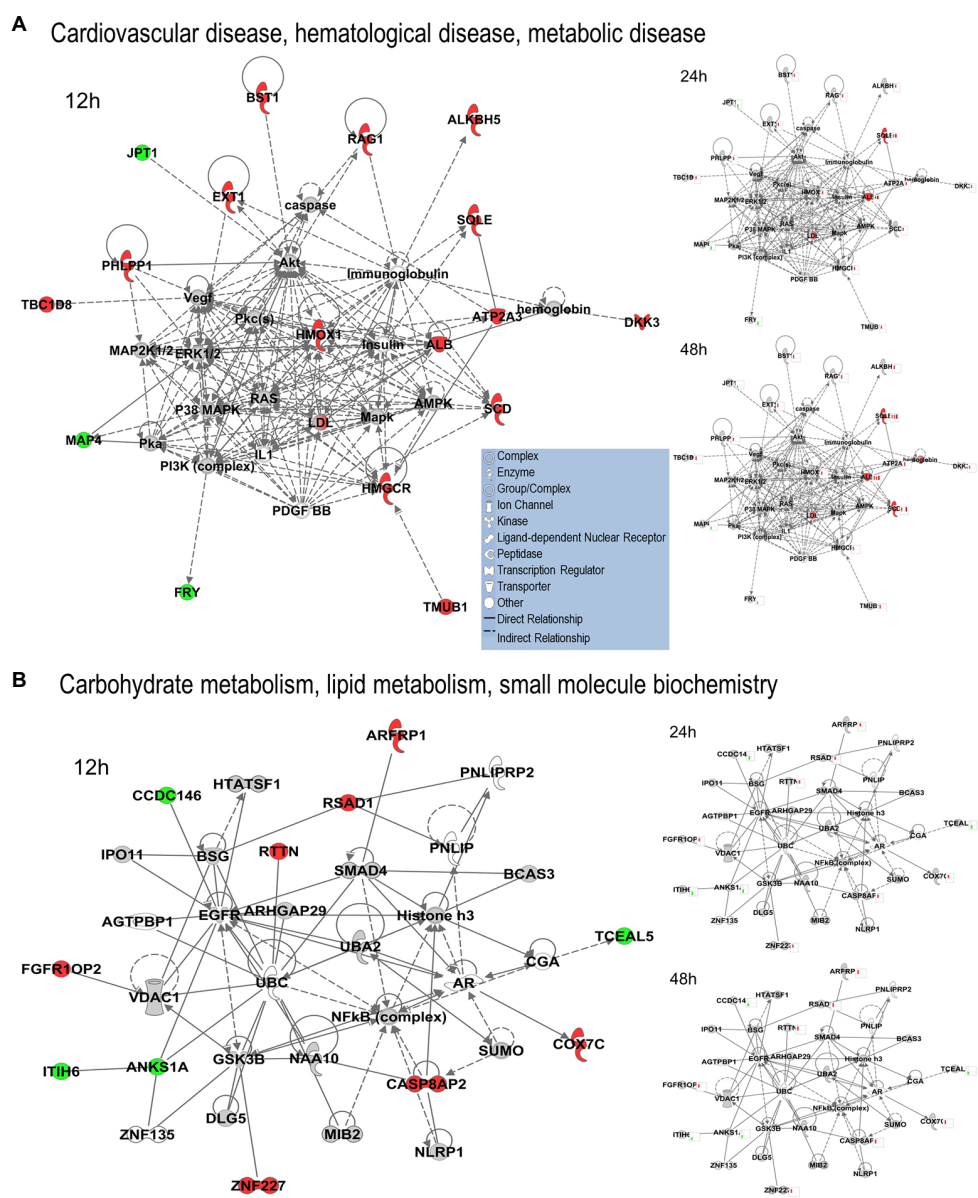
Top MRC-5 pathways affected by OC43 infection at 12  hpi. Proteins and their levels of dysregulation were uploaded into Ingenuity Pathway Analysis (IPA) and top pathways constructed. **(A)** The “cardiovascular disease, hematological disease, and metabolic disease” network. **(B)** The “carbohydrate metabolism, lipid metabolism, and small molecule biochemistry” network. Protein levels at 24 and at 48  hpi were overlaid onto these pathways (smaller diagrams at right). Upregulated proteins depicted in red, downregulated in green, insignificantly regulated in gray, direct interactions shown with solid lines, and indirect interactions depicted with dashed lines. Molecular types as indicated in key.

**Figure 3:**
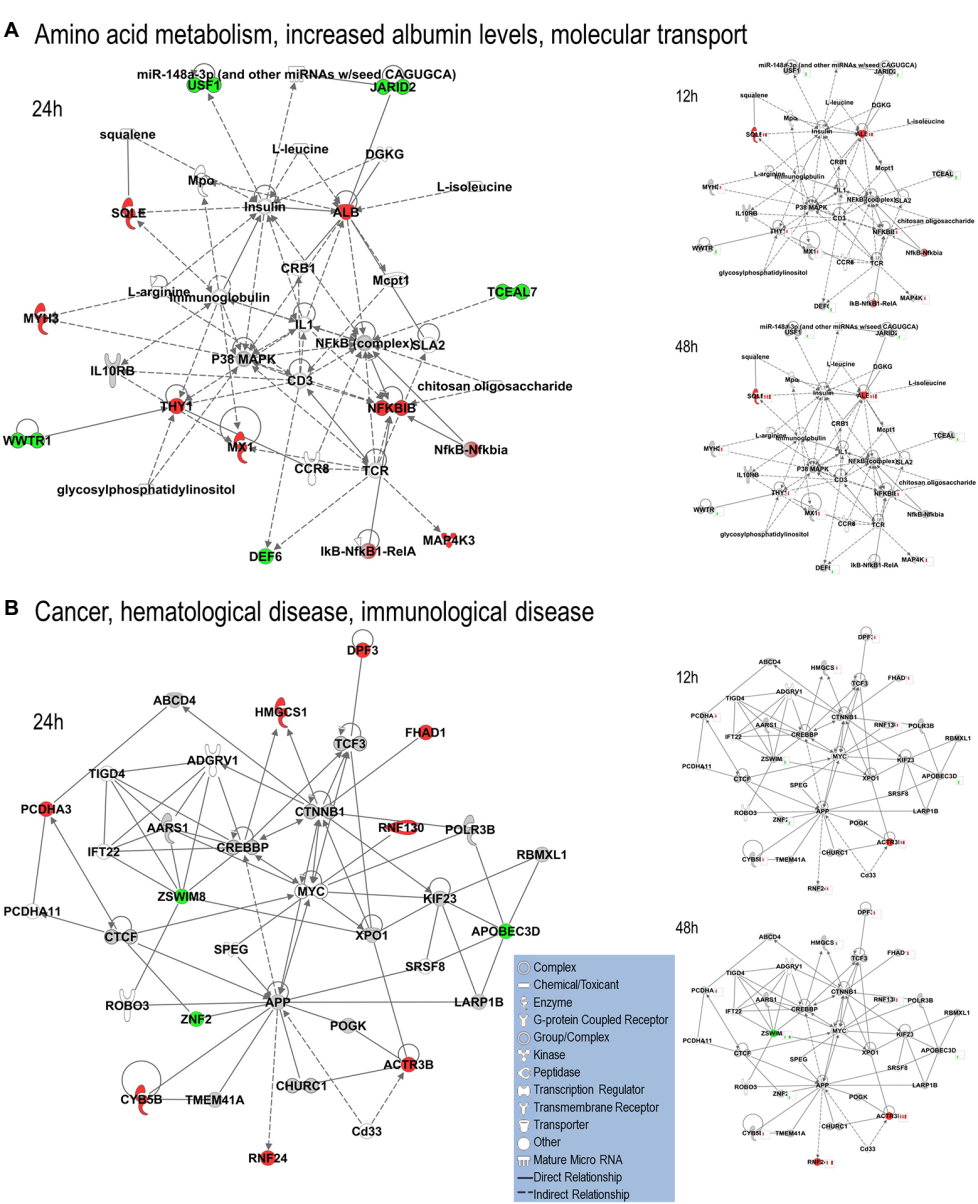
Top MRC-5 pathways affected by OC43 infection at 24  hpi. Proteins and their levels of dysregulation were uploaded into Ingenuity Pathway Analysis (IPA) and top pathways constructed. **(A)** The “amino acid metabolism, increased albumin levels, and molecular transport” network. **(B)** The “cancer, hematological disease and immunological disease” network. Protein levels at 12 and 48  hpi were overlaid onto these pathways (smaller diagrams at right). Upregulated proteins depicted in red, downregulated in green, insignificantly regulated in gray, direct interactions shown with solid lines and indirect interactions depicted with dashed lines. Molecular types as indicated in key.

**Figure 4:**
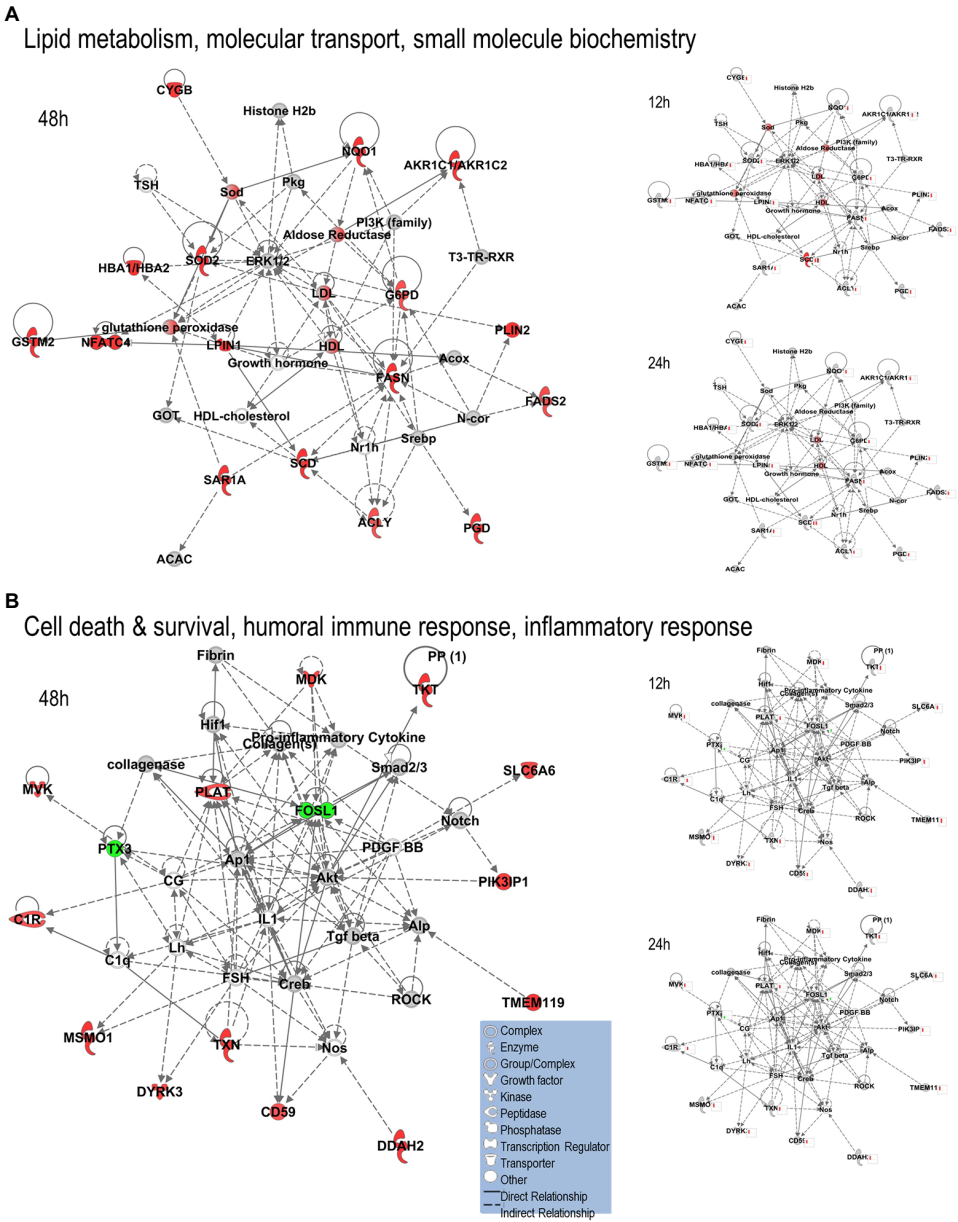
Top MRC-5 pathways affected by OC43 infection at 48  hpi. Proteins and their levels of dysregulation were uploaded into Ingenuity Pathway Analysis (IPA) and top pathways constructed. **(A)** The “lipid metabolism, molecular transport, and small molecule biochemistry” network. **(B)** The “cell death and survival, humoral immune response, and inflammatory response” network. Only the top two networks are shown. Additional networks, and their associated proteins, are listed in Supplementary Table S1. Protein levels at 12 and 24  hpi were overlaid onto these pathways (smaller diagrams at right). Upregulated proteins depicted in red, downregulated in green, insignificantly regulated in gray, direct interactions shown with solid lines, and indirect interactions depicted with dashed lines. Molecular types as indicated in key.

We uploaded the datasets of proteins and their levels of abundance into Ingenuity Pathway Analysis (IPA) to map networks predicted to be affected by OC43 infection and to observe temporal alterations in protein expressions. Only two networks were identified at 12 hpi with scores of 25 or more. One of these was the “cardiovascular disease, hematological disease, and metabolic disease” network (Figure 2A; Supplementary Table S1), with an IPA score of 43. This network was identified and built from 18 identified significantly dysregulated proteins. Fifteen of the proteins (ALB, ALKBH5, ATP2A3, BST1, DKK3, EXT1, HMGCR, HMOX1, LDL, PHLPP1, RAG1, SCD, SQLE, TBC1D8, and TMUB1) were significantly upregulated (Figure 2A, red) whereas only three (FRY, JPT1, and MAP4) were significantly downregulated (Figure 2A, green). The other network was “carbohydrate metabolism, lipid metabolism, and small molecule biochemistry” with a score of 25; seven proteins in this network were upregulated (ARFRP1, CASP8AP2, COX7C, FGFR1OP2, RSAD1, RTTN, and ZNF227), and four proteins (ANKS1A, CCDC146, ITIH6, and TCEAL5) were downregulated (Figure 2B). Several proteins within these networks were significantly upregulated at 12 hpi and remained so at later times post-infection [i.e., squalene monooxygenase (SQLE) and serum albumin (ALB)], whereas most proteins significantly dysregulated at 12 hpi were not significantly dysregulated at later time points (Figure 2). Similarly, two networks were identified at 24 hpi with IPA scores of 27 or more (Supplementary Table S1). One of these was “amino acid metabolism, increased albumin levels, and molecular transport” with a score of 30 (Figure 3A) and the other was “cancer, hematological disease, and immunological disease” with a score of 27 (Figure 3B). ALB and SQLE were upregulated in the “amino acid metabolism, increased albumin levels, and molecular transport” network at all time points, and actin-related protein 3B (ACTR3B) was upregulated in the “cancer, hematological disease, and immunological disease” network at all time points. Several proteins downregulated at 24 hpi [i.e., upstream stimulatory factor 1 (USF1) and zinc finger protein 2 (ZNF2)] were not dysregulated at earlier or later time points, whereas zinc finger SWIM domain-containing protein 8 (ZSWIM8) was downregulated at both 24 and 48 hpi. Myosin-3 (MYH3) and interferon-induced GTP-binding protein Mx1 (MX1) were upregulated at 24 hpi but not dysregulated at earlier or later time points, and RING finger protein 24 (RNF24) was upregulated at both 24 and 48 hpi (Figure 3). Four networks were identified at 48 hpi with scores of 22 or more and all contained 12 or more identified significantly dysregulated proteins. These networks were “lipid metabolism, molecular transport and small molecule biochemistry” with an IPA score of 32 (Figure 4A), “cell death and survival, humoral immune response, and inflammatory response” with a score of 30 (Figure 4B), “dermatological diseases and conditions, inflammatory response, and organismal injury and abnormalities” with a score of 27, and “metabolic disease, organismal injury, and abnormalities and renal and urological disease” with a score of 22. Several proteins in the “lipid metabolism, molecular transport, and small molecule biochemistry” network (i.e., HDL and LDL) were upregulated at all time points, whereas most dysregulated proteins [i.e., mitochondrial superoxide dismutase (SOD2), 6-phosphogluconate dehydrogenase, decarboxylating (PGD), midkine (MDK), and tissue-type plasminogen activator (PLAT)] were only dysregulated (up) at 48 hpi (Figure 4).

### Cellular diseases, functions, and pathways affected by OC43 infection

**Figure 5:**
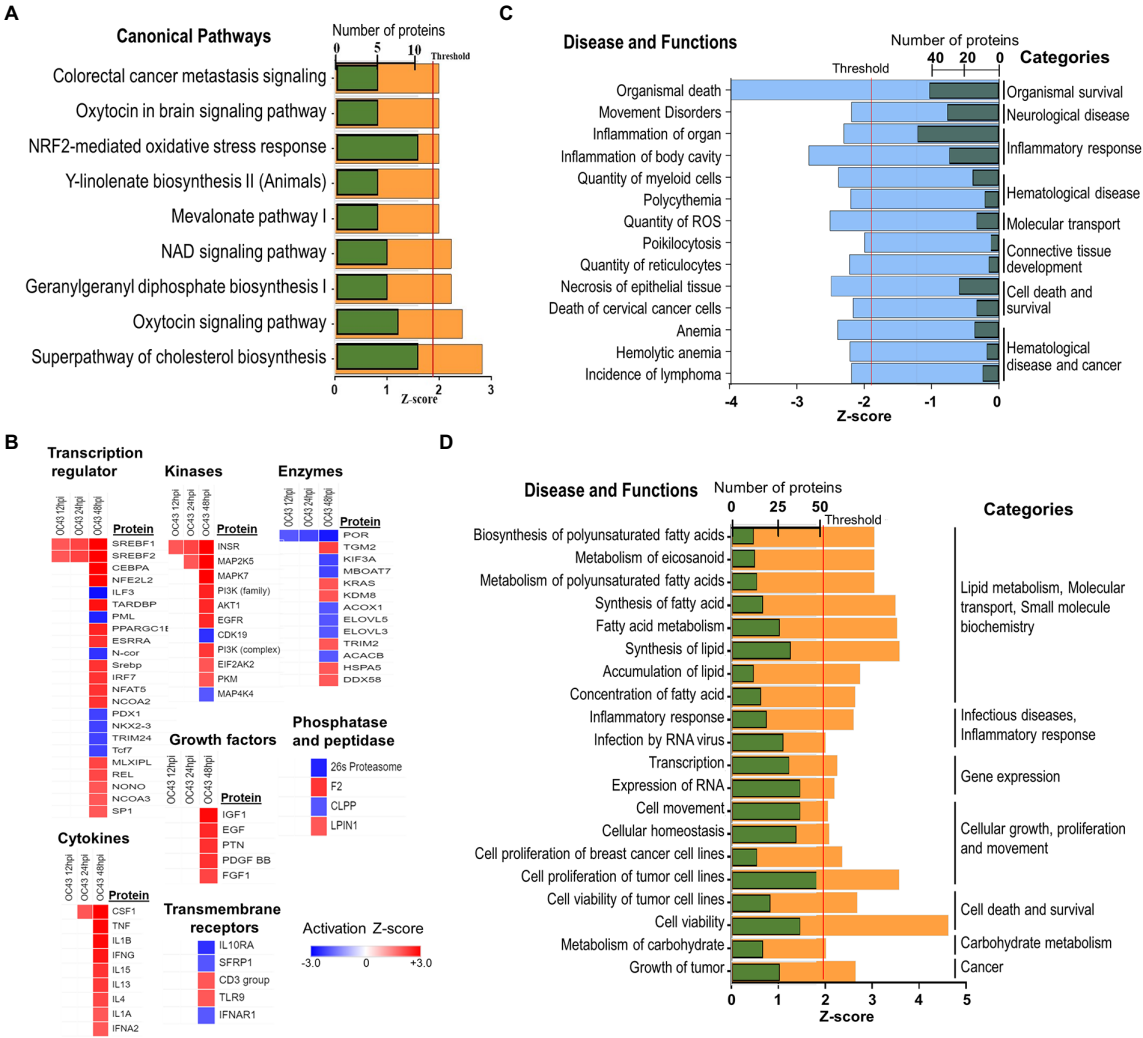
**(A)** IPA predicted activation and inhibition of canonical pathways by OC43 infection at 48  hpi. Green bars represent numbers of molecules significantly dysregulated in each pathway, with Z-score (orange) threshold set at +1.96σ. **(B)** IPA predicted activation and inhibition of upstream regulators, with heatmap of different types of upstream regulators indicated at various time post-infection. **(C)** Significantly inhibited diseases and functions. Green bars are numbers of molecules significantly dysregulated in each disease/function, with *Z*-score (blue) threshold set at −1.96σ. **(D)** Significantly activated diseases and functions. Green bars are numbers of molecules significantly dysregulated in each disease/function, with *Z*-score (orange) threshold set at +1.96σ. Specific proteins associated with each category are listed in Supplementary Table S2.

The identities and abundance levels of the significantly dysregulated proteins we observed also allowed IPA to predict various cellular processes, canonical pathways, and upstream regulators affected by OC43 infection (Figure 5). Some of the most significantly dysregulated canonical pathways were NRF2-mediated oxidative stress response, oxytocin signaling pathway, and superpathway of cholesterol biosynthesis, each with a predicted Z-score of >1.96σ (Figure 5A). Multiple upstream regulators of transcription, kinases, other enzymes, cytokines, and growth factors also were predicted to be affected (Figure 5B). For example, sterol regulatory element binding transcription factor 1 (SREBF1) and SREBF2 were predicted to be upregulated transcription factors at all time points post-infection whereas the vast majority of these regulators were predicted only to be up- or downregulated at late time points post-infection. The combined effects of the various upregulated and downregulated proteins we identified and measured led IPA to predict inhibition of 14 specific diseases/functions in eight categories (Figure 5C; Supplementary Table S2). For example, significant upregulation of 15 proteins (ACLY, ASS1, FADS2, FASN, G6PD, HSPB1, NAMPT, NQO1, PDCD4, PLIN2, SCD, SOD2, SQSTM1, TKT, and TXN, all of which were upregulated ≥1.33-fold), combined with significant downregulation of INCENP and TOP2A, both of which were downregulated ≥1.33-fold, combined with 18 other proteins being significantly dysregulated ≥1.25-fold (Supplementary Table S2), contributed to the identification of the “organismal death” disease/function in the “organismal survival” category. Similarly, the combined effects of the various upregulated and downregulated proteins we identified and measured led IPA to predict activation of more than 20 specific diseases/functions in more than eight categories (Figure 5D; Supplementary Table S2). Many of these significantly dysregulated proteins play important roles in several disease/functional processes. For example, upregulation of NQO1 (NAD(P)H dehydrogenase [quinone] 1) contributed to prediction that lymphoma incidence, anemia, ROS quantity, and other processes were inhibited, whereas carbohydrate metabolism, nitric oxide synthesis, RNA virus infection, fatty acid metabolism, and other processes were predicted to be activated (Supplementary Table S2).

## Discussion

We used non-biased quantitative mass spectrometry to measure more than 8,300 cellular proteins across three time points. This led to the identification of >1,500 significantly dysregulated proteins at any time point (*p* < 0.05). This strategy has been useful in identifying alterations induced in cellular proteins after infection by numerous viruses, including HIV (Greenwood et al., 2016), influenza viruses (Vester et al., 2009; Coombs et al., 2010; Ranadheera et al., 2018), herpesvirus (Berard et al., 2015a; Wan et al., 2019), and Zika virus (Xin et al., 2017; Srivastava et al., 2020; Rashid et al., 2022). Some of the proteins considered significantly dysregulated by either T-test or Z-score analysis were only altered in quantity by as little as 5%. For example, 1,510 proteins were significantly dysregulated at any time point, but almost 200 of them were dysregulated 10% or less. Numerous studies have used various fold-change cutoffs ranging from as low as 5% to as much as 2-fold or more. However, we noted that only 11 MRC-5 proteins were dysregulated by OC43 2-fold or more, far too few for meaningful downstream pathway analyses. Thus, we chose a fold-change cutoff of 1.33 for IPA pathway analyses to have a reasonable number to analyze while still maintaining some stringency. As seen in several other studies (Coombs et al., 2010; Berard et al., 2015b; Glover et al., 2019; Rashid et al., 2022), the numbers and types of affected proteins and networks increased as time progressed. At the earliest time point examined (12 hpi), 27 proteins were significantly upregulated ≥1.33-fold and eight were significantly downregulated ≥1.33-fold (Table 1). These proteins led to IPA-predicted construction of two networks; the cardiovascular disease, hematological disease, and metabolic disease network (Figure 2A); and the carbohydrate metabolism, lipid metabolism, and small molecule biochemistry network (Figure 2B). While a few of the upregulated proteins [i.e., ACTR3B, Acyl-CoA desaturase (SCD) and SQLE] also were upregulated at later time points (Figure 2; Table 2), the majority of the proteins either upregulated or downregulated at 12 hpi were not dysregulated at later times, implying they are altered soon after OC43 infection. Fewer proteins (14 up and 9 down) were significantly dysregulated at 24 hpi and these also fit into two IPA-predicted networks: the amino acid metabolism, increased albumin levels, and molecular transport network (Figure 3A) and the cancer, hematological disease, and immunological disease network (Figure 3B). None of the proteins significantly upregulated ≥1.33-fold at 24 hpi (except for ACTR3B, SQLE, and RNF24) were significantly upregulated at other time points, and only ZSWIM8, which was significantly downregulated was so at 24 and 48 hpi. Thus, the majority of 24 hpi dysregulated proteins and networks appear to be transiently affected. Many more proteins and network pathways were predicted by IPA to be affected by 48 hpi (Tables 1, 2; Figure 4). These were the lipid metabolism, molecular transport, and small molecule biochemistry network (Figure 4A), the cell death and survival, humoral immune response, and inflammatory response network (Figure 4B), the dermatological diseases and conditions, inflammatory response and organismal injury and abnormalities network, and the metabolic disease, organismal injury, and abnormalities, and renal and urological disease network.

Some of the most highly dysregulated proteins identified were ACTR3, ATP2A3, CASP8AP2, CEP128, FHAD1, HERV-K104, MYH3, RSAD1, and SAMD8, all of which were upregulated, and ZSWIM8, which was downregulated. ACTR3B (actin-related protein 3B) protein expression has been linked to colorectal cancer invasion and proliferation (Yu and Zhang, 2020), it is part of a complex with CAPG and CD3D that interacts with several HIV proteins (Zhang et al., 2019) and its expression aids SARS-CoV-2 binding and intracellular processing (Kalejaiye et al., 2022). Sarcoplasmic/endoplasmic reticulum calcium ATPase 3 (ATP2A3) is involved in numerous cancers (Korosec et al., 2009; Flores-Peredo et al., 2017; Izquierdo-Torres et al., 2017), but we are not aware of any association with virus infection. CASP8-associated protein 2 (CASP8AP2) is involved in apoptosis and autophagy (Wu et al., 2021) and in leukemia (Mei et al., 2017; Dos Santos et al., 2018). Centrosomal protein of 128 kDa (CEP128) plays a role in bladder cancer (Wu et al., 2018) and autoimmune thyroid disease (Wang et al., 2019). Forkhead-associated domain-containing protein 1 (FHAD1) has been identified as a possible biomarker for prostate cancer (Zhao et al., 2017). Mutations in myosin heavy chain 3 (MYH3) are associated with numerous autosomal-dominant defects (Chong et al., 2015). Radical S-adenosyl methionine domain-containing protein 1, mitochondrial (RSAD1) is a heme chaperone that inserts heme into respiratory protein targets (Haskamp et al., 2018). Many of the above-noted proteins have not yet been associated with viral infection, but Sterile alpha motif domain-containing 9 (SAMD9) has antiviral properties (Liu and McFadden, 2015; Mekhedov et al., 2017). Zinc finger SWIM domain-containing protein 8 (ZSWIM) mediates target-directed mRNA degradation (Han et al., 2020; Shi et al., 2020).

Bioinformatic analyses to place the large amount of data generated in studies such as this provides useful information that must be carefully considered. For example, IPA has a bias toward cancer processes (Edlow et al., 2015). Thus, despite apparent identification of specific diseases such as “cell proliferation of breast cancer cell lines” and “cell movement of cervical cancer cell lines” (Figure 5D; Supplementary Table S2), these processes are likely irrelevant in the diploid MRC-5 lung cells we used. However, many of the IPA-predicted diseases, functions, and networks are highly relevant within the context of viral-infected lung cells. For example, six proteins: hemoglobin subunit alpha (HBA1/HBA2), interferon-induced 35 kDa protein (IFI35), nicotinamide phosphoribosyltransferase (NAMPT), NQO1, poly [ADP-ribose] polymerase 9 (PARP9), and tripartite motif-containing protein 5 (TRIM5) were significantly upregulated ≥1.33-fold, DNA topoisomerase 2-alpha (TOP2A) was significantly downregulated ≥1.33-fold, and an additional 20 proteins were significantly dysregulated ≥1.25-fold (Supplementary Table S2), contributing to identification of the “infection by RNA virus” disease function. Since OC43 and other CoV are RNA viruses, downregulation of TOP2A, and upregulation of various RNA metabolic enzymes likely makes sense. Likewise, many viruses induce interferon-stimulated and interferon-response genes. Similarly, many of the significantly dysregulated proteins [i.e., ATP-citrate synthase (ACLY), fatty acid synthase (FASN), glucose-6-phosphate 1-dehydrogenase (G6PD), perilipin-2 (PLIN2), and others] are involved in activation of small molecule biochemistry and lipid metabolism. These processes are expected to be activated during infection by an enveloped virus such as CoV since the virus takes over cellular metabolic processes and reorganizes cellular lipids and fatty acids to promote synthesis and maturation of hundreds of copies of itself in each cell.

**Figure 6:**
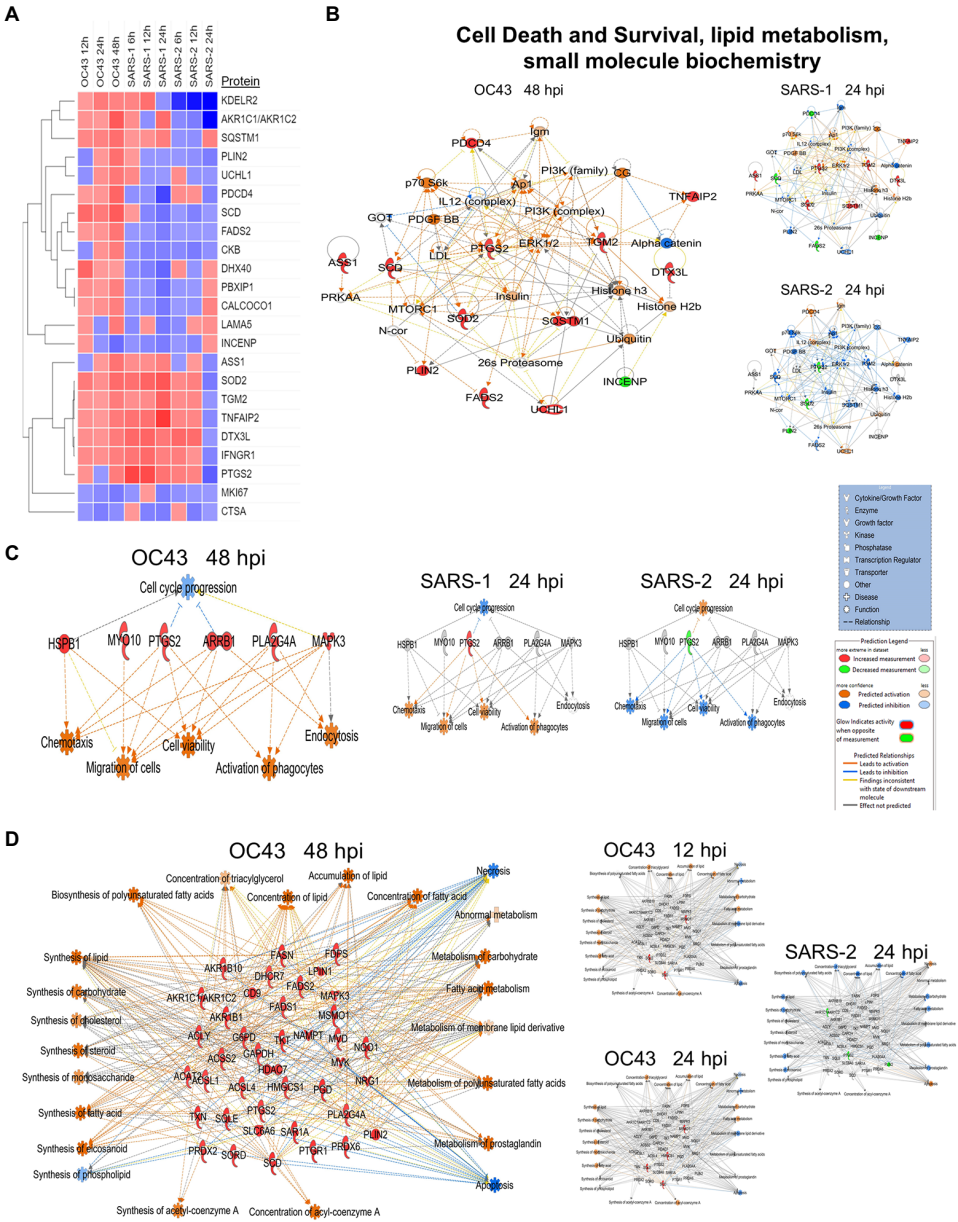
**(A)** Proteins significantly dysregulated by OC43, SARS-1, and SARS-CoV-2 (SARS-2). **(B)** Proteins in “A” are primarily associated with the” cell death and survival” network. **(C)** Differential oxytocin signaling pathway. **(D)** Differential expression of proteins associated with synthesis and metabolism of lipid and carbohydrate, cellular lipid concentration, and energy generation. Red and green colors represent up- and downregulation of proteins, respectively, whereas orange and blue depict pathway activation and inhibition, respectively.

**Figure 7:**
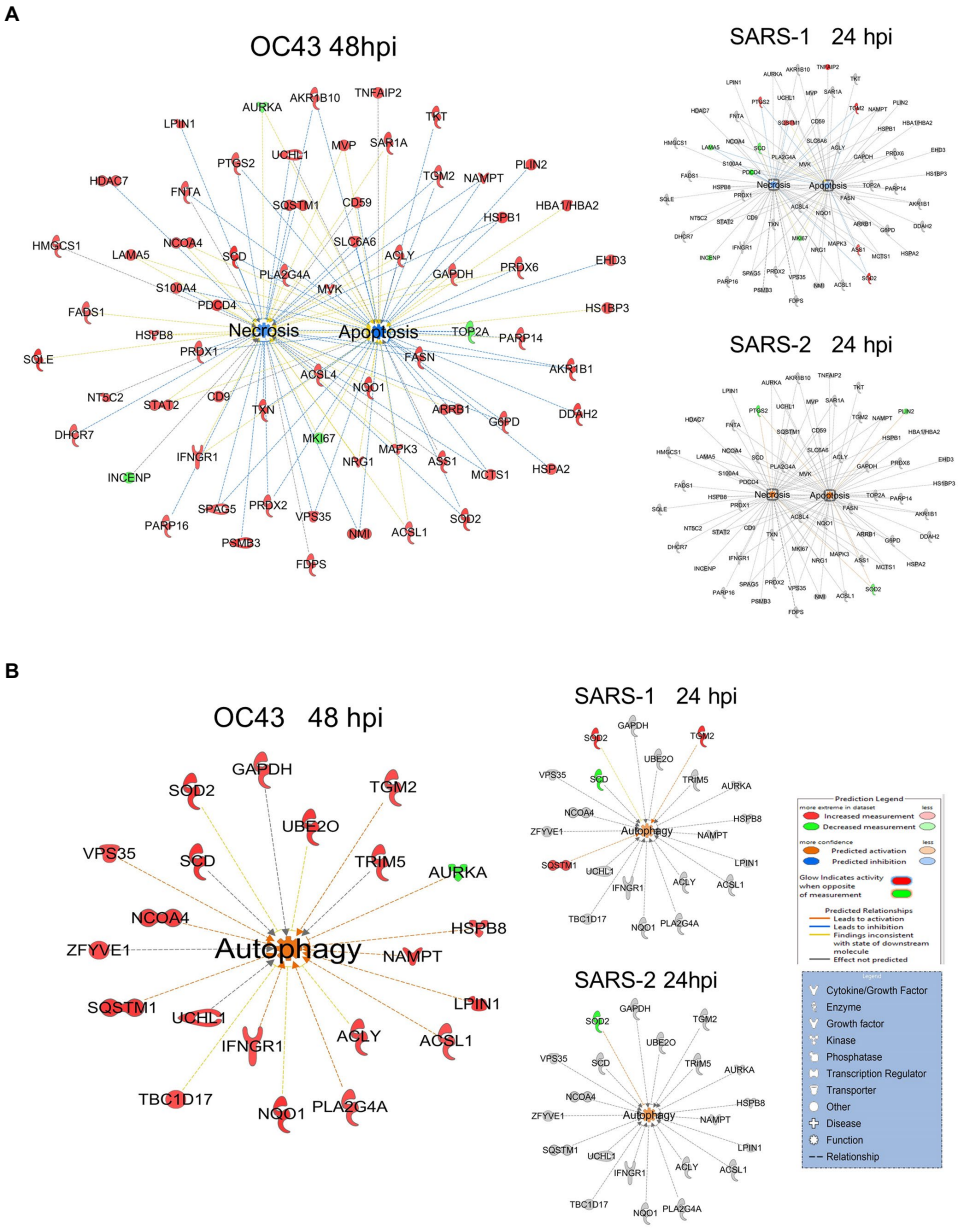
**(A)** OC43 and SARS-1 infection inhibits apoptosis and necrosis, but SARS-CoV-2 (SARS-2) infection activates apoptosis and necrosis. **(B)** All viruses activate autophagy. Red and green colors represent up- and downregulation of proteins, respectively.

A primary purpose for performing these studies was to identify cellular proteins, pathways, and processes that are similar or different between the CL2-level OC43 coronavirus that causes mild cold-like symptoms, and more pathogenic coronaviruses such as SARS1 and SARS-CoV-2. Similar non-biased proteomic studies have been reported for the more pathogenic coronaviruses, including by Bojkova and colleagues who examined SARS-CoV-2-induced alterations in human intestinal Caco-2 cells (Bojkova et al., 2020), Bouhaddou et al. who examined phosphoproteomic alterations in African green monkey VeroE6 cells (Bouhaddou et al., 2020), Hekman and colleagues who examined SARS-CoV-2-induced alterations in human alveolar type 2 cells (Hekman et al., 2020), and Stukalov et al. who examined SARS1- and SARS-CoV-2-induced protein alterations in human lung A549 cells (Stukalov et al., 2021). Therefore, we compared our results to those of Stukalov and colleagues who measured SARS1- and SARS-CoV-2-induced alterations in another human lung cell type. Several proteins (i.e., FADS2 – fatty acid desaturase 2; CKB – creatine kinase B) were differentially regulated, being upregulated by OC43 at all tested time points, and being downregulated by SARS1 and SARS-CoV-2 at all tested time points (Figure 6A). These differences in protein dysregulation led to substantial differences in predicted pathways, particularly in comparing the milder OD43 to the more pathogenic SARS-CoV-2. For example, numerous proteins in the cell death and survival, lipid metabolism, and small molecule biochemistry pathways were upregulated, whereas many of these same proteins were downregulated by SARS-CoV-2 (Figures 6B–D). Interestingly, SARS1 had similar effects upon most of these proteins as OC43, potentially reflecting that while SARS1 is pathogenic, it appears to be less so than SARS-CoV-2. Similarly, OC43 and SARS1 infection were predicted to inhibit apoptosis and necrosis, whereas infection by the more pathogenic SARS-CoV-2 was predicted to activate necrosis and apoptosis (Figure 7A). All viruses were predicted to activate autophagy (Figure 7B). Apoptosis and necroptosis (the regulated form of necrosis), the two major processes of programmed cell death (PCD), protect cells against intracellular infection (Doerflinger et al., 2020). They can also trigger innate and adaptive immunological responses, as well as inflammation (Bedoui et al., 2020). PCD can help the host by eliminating virus-infected cells and initiating immune response, but uncontrolled activation of the pathways can cause severe tissue damage. Recent studies have found that activation of apoptosis and necroptosis is associated with the severe outcome of COVID-19 disease (Lee et al., 2020; Bader et al., 2022; Da Silva et al., 2022). However, the apoptosis and necroptosis pathways were inhibited during OC43 infection (Figure 7A), causing a milder type of disease. Thus, PCD pathways could be a potential target for therapeutic drug development for the treatment of severe COVID-19 disease. However, the autophagy pathway plays a critical role in SARS-CoV-2 pathogenesis (Gassen et al., 2021; Maity and Saha, 2021; Vanhook, 2021). As all three coronavirus strains activated autophagy, this pathway could be a universal target for antiviral development.

**Figure 8:**
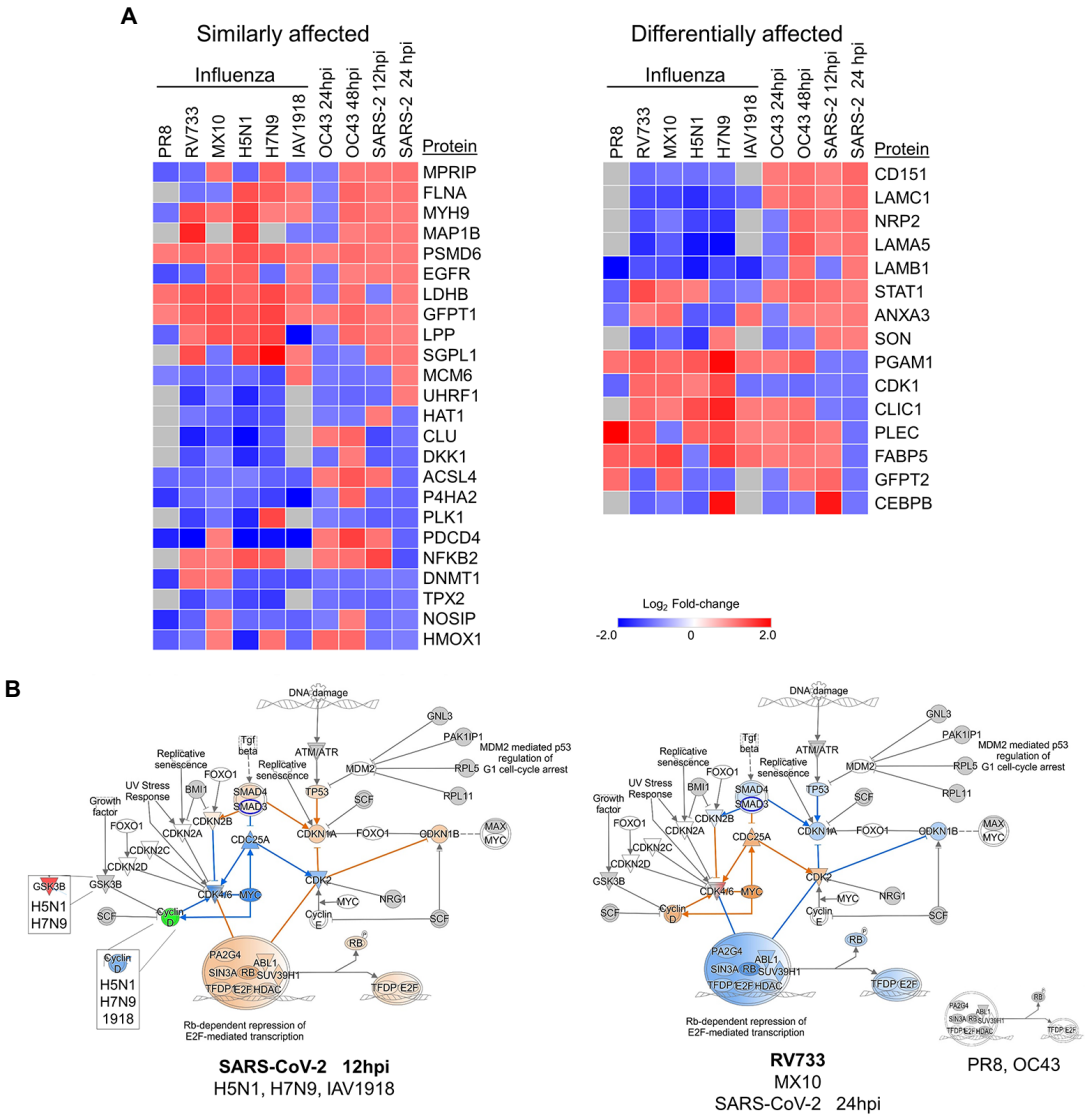
**(A)** Various influenza A viruses and coronaviruses (CoV) have similar (left) and dissimilar (right) effects on several human lung cell proteins. **(B)** The highly pathogenic IAV and CoV (left) activate Rb-dependent repression of E2F-mediated transcription whereas lower pathogenic IAV and CoV (right) either have no effect (PR8 and OC43) or inhibit the pathway. Red and green colors represent up- and downregulation of proteins, respectively and orange and blue depict activation and inhibition, respectively.

We (Coombs et al., 2010, 2017; Simon et al., 2015; Ranadheera et al., 2018) and others (Liu et al., 2008; Vester et al., 2009; Dove et al., 2012) also have examined quantitative proteomic alterations induced by various influenza viruses, another common respiratory virus also responsible for numerous recent pandemics. Therefore, we compared these multiple datasets to identify common and virus-specific proteomic signatures (Figure 8). Numerous proteins involved in the cell cycle, cytokine signaling, DNA replication, and anti-inflammatory responses were generally similarly affected by virtually all IAV and CoV tested, irrespective of whether the IAV and CoV were mildly or highly pathogenic (Figure 8A, left). For example, GFPT1 (glucosamine—fructose-6-phosphate aminotransferase isomerizing 1) and PSMD6 (proteasome 26S subunit, non-ATPase 6) were upregulated by all tested IAV and CoV, and NOSIP (nitric oxide synthase interacting protein) and P4HA2 (prolyl 4-hydroxylase subunit alpha-2) were downregulated by most viruses (with the relatively milder IAV pdm09 and CoV OC43 being lone exceptions). These results suggest some common cellular pathways that might represent targets for a “universal” anti-viral strategy. Conversely, levels of proteins involved in necrosis, protein metabolism, ECM regulation, and signal transduction were generally differentially affected by IAV and by CoV (Figure 8B, right). For example, CD151 (cluster of differentiation 151, a tetraspanin membrane protein) and LAMC1 (laminin subunit gamma 1, a constituent of basement membranes) were upregulated by all CoV but unaffected or downregulated by all IAV. CLIC1 (chloride intracellular channel protein 1, which regulates important cellular processes such as maintenance of intracellular pH, regulation of cell volume, stabilization of cell membrane potential, and transepithelial transport) and PGAM1 (phosphoglycerate mutase 1, which is involved in protein kinase binding and Menkes Disease and myoglobinuria) were downregulated only by the more pathogenic SARS-COV-2 but upregulated by virtually all other viruses. These results indicate CoV and IAV affect these tested lung cells in different ways.

The quantitative differences in protein levels in CoV- and IAV-infected lung cells also led to IPA predicting significant differences in Rb-dependent repression of E2F-mediated transcription, with the more pathogenic CoV and IAV predicted to activate this checkpoint whereas less pathogenic CoV and IAV either had no effect or were predicted to inhibit this checkpoint (Figure 8B). In general, the less pathogenic PR8, RV733, pdm09, and OC43 viruses inhibited TP53, CDKN1A, CDKN1B, and SMAD4 and activated CDC25A, Cyclin D, CDK2, and MYC, whereas the more pathogenic H5N1, H7N9, 1918 and SARS-CoV-2 had the opposite effect upon these molecules. The retinoblastoma-dependent repression of E2F-mediated transcription checkpoint is highly involved in the cell cycle and in cancer. Thus, it plays an intimate role in cell death and survival. Intriguingly, SARS-CoV-2 appeared to have a temporal effect on this pathway, predicted to activate it early in infection but to inhibit it later in infection. Thus, the G1/S checkpoint might be an attractive anti-viral target, and drug repurposing to overcome the activation caused by the pathogenic viruses may attenuate disease.

One of the limitations of these IAV and CoV comparative studies relates to cell lines used. The A549 cells are transformed adenocarcinoma human lung cells amenable to infection by many viruses and were used by us in previous IAV studies (Coombs et al., 2010, 2017; Simon et al., 2015; Ranadheera et al., 2018). This cell type also was used by Stukalov and colleagues to examine SARS-CoV-2-induced dysregulation (Bojkova et al., 2020). Unfortunately, we were unable to grow OC43 in these cells. OC43 grows well in human diploid MRC-5 cells so these were used. These different cell types could explain some of the differences seen in how the various viruses induced proteomic responses, but despite this, it remains remarkable that the Rb-dependent repression of E2F-mediated transcription checkpoint appears to distinguish high-pathogenicity viruses from low-pathogenicity viruses.

## Conclusion

Non-biased TMT-based proteomics of OC43-infected human lung cells identified large numbers of dysregulated cellular proteins. These proteins are involved in a plethora of cellular processes, including cell death and survival, humoral immune responses, inflammatory responses, lipid metabolism, molecular transport, and small molecule biochemistry. Comparisons of these data with our, and others, data using similar methods to examine influenza virus- and SARS-CoV-2-induced cellular protein dysregulation revealed numerous proteins and cell pathways affected similarly by both types of viruses as well as virus-specific protein and pathway alterations. Interestingly, analyses of IAV and CoV of different pathogenicities suggested that the more pathogenic viruses could activate the Rb-dependent repression of E2F-mediated transcription checkpoint, whereas less pathogenic viruses either had no effect or inhibited this checkpoint. Furthermore, while many of these studies were performed in cell culture models, it would be worthwhile for follow-up studies to examine the roles of these proteins and pathways in natural infections.

## Data availability statement

The datasets presented in this study can be found in online repositories. The names of the repository/repositories and accession number(s) can be found at: University of California, San Diego's MassIVE archive -MSV000089582.

## Author contributions

KC: conceptualization, writing—original draft preparation, supervision, project administration, and funding acquisition. YL and VS: methodology. VS: software. M-uR, KG, and KC: validation, formal analysis, and investigation. M-uR, KG, YL, VS, and KC: writing—review and editing. M-uR and KC: visualization. All authors contributed to the article and approved the submitted version.

## Funding

The Canadian Institutes of Health Research, grants MOP-106713 and VR3-172641 to KC funded this research. The Canadian Foundation for Innovation provided funding and support for the mass spectrometry instruments. Research Manitoba Studentships supported M-uR and KG.

## Conflict of interest

The authors declare that the research was conducted in the absence of any commercial or financial relationships that could be construed as a potential conflict of interest.

## Publisher’s note

All claims expressed in this article are solely those of the authors and do not necessarily represent those of their affiliated organizations, or those of the publisher, the editors and the reviewers. Any product that may be evaluated in this article, or claim that may be made by its manufacturer, is not guaranteed or endorsed by the publisher.

## Footnotes

^1^ massive.ucsd.edu

## References

[ref1] Bader S. M. Cooney J. P. Pellegrini M. Doerflinger M. (2022). Programmed cell death: the pathways to severe COVID-19? Biochem. J. 479, 609–628. doi: 10.1042/BCJ20210602, PMID: 35244141 PMC9022977

[ref2] Bedoui S. Herold M. J. Strasser A. (2020). Emerging connectivity of programmed cell death pathways and its physiological implications. Nat. Rev. Mol. Cell Biol. 21, 678–695. doi: 10.1038/s41580-020-0270-8, PMID: 32873928

[ref3] Berard A. R. Coombs K. M. Severini A. (2015a). Quantification of the host response proteome after herpes simplex 1 virus infection. J. Proteome Res. 14, 2121–2142. doi: 10.1021/pr5012284, PMID: 25815715

[ref4] Berard A. R. Severini A. Coombs K. M. (2015b). Comparative proteomic analyses of two reovirus T3D subtypes and comparison to T1L identifies multiple novel proteins in key cellular pathogenic pathways. Proteomics 15, 2113–2135. doi: 10.1002/pmic.201400602, PMID: 25900405

[ref5] Bojkova D. Klann K. Koch B. Widera M. Krause D. Ciesek S.. (2020). Proteomics of SARS-CoV-2-infected host cells reveals therapy targets. Nature 583, 469–472. doi: 10.1038/s41586-020-2332-7, PMID: 32408336 PMC7616921

[ref6] Bouhaddou M. Memon D. Meyer B. White K. M. Rezelj V. V. Marrero M. C.. (2020). The global phosphorylation landscape of SARS-CoV-2 infection. Cells 182, 685–712.e19. doi: 10.1016/j.cell.2020.06.034, PMID: 32645325 PMC7321036

[ref7] Chong J. X. Burrage L. C. Beck A. E. Marvin C. T. Mcmillin M. J. Shively K. M.. (2015). Autosomal-dominant multiple pterygium syndrome is caused by mutations in MYH3. Am. J. Hum. Genet. 96, 841–849. doi: 10.1016/j.ajhg.2015.04.004, PMID: 25957469 PMC4570285

[ref8] Coombs K. M. Berard A. Xu W. Krokhin O. Meng X. Cortens J. P.. (2010). Quantitative proteomic analyses of influenza virus-infected cultured human lung cells. J. Virol. 84, 10888–10906. doi: 10.1128/JVI.00431-10, PMID: 20702633 PMC2950599

[ref9] Coombs K. M. Zhang X. L. Hu P. (2017). Aptamer profiling of influenza virus-infected cells highlights dysregulated receptor signaling.

[ref10] Da Silva M. M. De Lucena A. S. L. Paiva S. D. L. De Carvalho V. M. F. De Oliveira P. S. S. Da Rosa M. M.. (2022). Cell death mechanisms involved in cell injury caused by SARS-CoV-2. Rev. Med. Virol. 32:e2292. doi: 10.1002/rmv.2292 34590761 PMC8646768

[ref11] Delany I. Rappuoli R. De Gregorio E. (2014). Vaccines for the 21st century. EMBO Mol. Med. 6, 708–720. doi: 10.1002/emmm.201403876, PMID: 24803000 PMC4203350

[ref12] Doerflinger M. Deng Y. X. Whitney P. Salvamoser R. Engel S. Kueh A. J.. (2020). Flexible usage and interconnectivity of diverse cell death pathways protect against intracellular infection. Immunity 53, 533–547.e7. doi: 10.1016/j.immuni.2020.07.004, PMID: 32735843 PMC7500851

[ref13] Dos Santos D. M. C. Eilers J. Vizcaino A. S. Orlova E. Zimmermann M. Stanulla M.. (2018). MAP3K7 is recurrently deleted in pediatric T-lymphoblastic leukemia and affects cell proliferation independently of NF-kappa B. BMC Cancer 18:633. doi: 10.1186/s12885-018-4525-0 29914415 PMC6006985

[ref14] Dove B. K. Surtees R. Bean T. J. Munday D. Wise H. M. Digard P.. (2012). A quantitative proteomic analysis of lung epithelial (A549) cells infected with 2009 pandemic influenza a virus using stable isotope labelling with amino acids in cell culture. Proteomics 12, 1431–1436. doi: 10.1002/pmic.201100470, PMID: 22585751

[ref15] Edlow A. Slonim D. Wick H. Hui L. S. Bianchi D. (2015). The pathway not taken: understanding 'omics data in the perinatal context. Am. J. Obstet. Gynecol. 212, S62–S63. doi: 10.1016/j.ajog.2014.10.135 PMC4485545 25772209

[ref16] Flores-Peredo L. Rodriguez G. Zarain-Herzberg A. (2017). Induction of cell differentiation activates transcription of the Sarco/endoplasmic reticulum calcium-ATPase 3 gene (ATP2A3) in gastric and colon cancer cells. Mol. Carcinog. 56, 735–750. doi: 10.1002/mc.22529, PMID: 27433831

[ref17] Gassen N. C. Papies J. Bajaj T. Emanuel J. Dethloff F. Chua R. L.. (2021). SARS-CoV-2-mediated dysregulation of metabolism and autophagy uncovers host-targeting antivirals. Nat. Commun. 12:3818. doi: 10.1038/s41467-021-24007-w 34155207 PMC8217552

[ref18] Glover K. K. M. Gao A. Zahedi-Amiri A. Coombs K. M. (2019). Vero cell proteomic changes induced by Zika virus infection. Proteomics 19:e1800309. doi: 10.1002/pmic.201800309 30578658

[ref19] Greenwood E. J. D. Matheson N. J. Wals K. Van Den Boomen D. J. H. Antrobus R. Williamson J. C.. (2016). Temporal proteomic analysis of HIV infection reveals remodelling of the host phosphoproteome by lentiviral Vif variants. elife 5:e18296. doi: 10.7554/eLife.18296, PMID: 27690223 PMC5085607

[ref20] Han J. Lavigne C. A. Jones B. T. Zhang H. Gillett F. Mendell J. T. (2020). A ubiquitin ligase mediates target-directed microRNA decay independently of tailing and trimming. Science 370:eabc9546. doi: 10.1126/science.abc9546, PMID: 33184234 PMC8177725

[ref21] Haskamp V. Karrie S. Mingers T. Barthels S. Alberge F. Magalon A.. (2018). The radical SAM protein HemW is a heme chaperone. J. Biol. Chem. 293, 2558–2572. doi: 10.1074/jbc.RA117.000229, PMID: 29282292 PMC5818191

[ref22] Hekman R. M. Hume A. J. Goel R. K. Abo K. M. Huang J. Blum B. C.. (2020). Actionable cytopathogenic host responses of human alveolar type 2 cells to SARS-CoV-2. Mol. Cell 80, 1104–1122.e9. doi: 10.1016/j.molcel.2020.11.028, PMID: 33259812 PMC7674017

[ref23] Izquierdo-Torres E. Rodriguez G. Meneses-Morales I. Zarain-Herzberg A. (2017). ATP2A3 gene as an important player for resveratrol anticancer activity in breast cancer cells. Mol. Carcinog. 56, 1703–1711. doi: 10.1002/mc.22625, PMID: 28150875

[ref24] Kalejaiye T. D. Bhattacharya R. Burt M. A. Travieso T. Okafor A. E. Mou X.. (2022). BSG/CD147 and ACE2 receptors facilitate SARS-CoV-2 infection of human iPS cell-derived kidney podocytes. bioRxiv [Preprint]. doi: 10.1101/2021.11.16.468893 PMC9065256 35517495

[ref25] Korosec B. Glavac D. Volavsek M. Ravnik-Glavac M. (2009). ATP2A3 gene is involved in cancer susceptibility. Cancer Genet. Cytogenet. 188, 88–94. doi: 10.1016/j.cancergencyto.2008.10.007, PMID: 19100511

[ref26] Lambert F. Jacomy H. Marceau G. Talbot P. J. (2008). “Titration of human coronaviruses, HCoV-229E and HCoV-OC43, by an indirect immunoperoxidase assay, SARS- and other coronaviruses,” in Methods and Molecular Biology. eds. Cavanagh D. (Totowa, NJ: Humana Press). 10.1007/978-1-59745-181-9_8 PMC7121483 19057861

[ref27] Lee S. Channappanavar R. Kanneganti T. D. (2020). Coronaviruses: innate immunity, inflammasome activation, inflammatory cell death, and cytokines. Trends Immunol. 41, 1083–1099. doi: 10.1016/j.it.2020.10.005, PMID: 33153908 PMC7561287

[ref28] Liu J. Mcfadden G. (2015). SAMD9 is an innate antiviral host factor with stress response properties that can be antagonized by poxviruses. J. Virol. 89, 1925–1931. doi: 10.1128/JVI.02262-14, PMID: 25428864 PMC4300762

[ref29] Liu N. Song W. J. Wang P. Lee K. C. Chan W. Chen H. L.. (2008). Proteomics analysis of differential expression of cellular proteins in response to avian H9N2 virus infection in human cells. Proteomics 8, 1851–1858. doi: 10.1002/pmic.200700757, PMID: 18398875

[ref30] Maity S. Saha A. (2021). Therapeutic potential of exploiting autophagy cascade against coronavirus infection. Front. Microbiol. 12:675419. doi: 10.3389/fmicb.2021.675419, PMID: 34054782 PMC8160449

[ref31] Mei Y. Y. Li Z. G. Zhang Y. Zhang W. L. Hu H. M. Zhang P. W.. (2017). Low miR-210 and CASP8AP2 expression is associated with a poor outcome in pediatric acute lymphoblastic leukemia. Oncol. Lett. 14, 8072–8077. doi: 10.3892/ol.2017.7229, PMID: 29250188 PMC5730016

[ref32] Mekhedov S. L. Makarova K. S. Koonin E. V. (2017). The complex domain architecture of SAMD9 family proteins, predicted STAND-like NTPases, suggests new links to inflammation and apoptosis. Biol. Direct 12:13. doi: 10.1186/s13062-017-0185-2, PMID: 28545555 PMC5445408

[ref33] Nypaver C. Dehlinger C. Carter C. (2021). Influenza and influenza vaccine: A review. J. Midwifery Womens Health 66, 45–53. doi: 10.1111/jmwh.13203, PMID: 33522695 PMC8014756

[ref34] Ranadheera C. Coombs K. M. Kobasa D. (2018). Comprehending a killer: the Akt/mTOR signaling pathways are temporally high-jacked by the highly pathogenic 1918 influenza virus. EBioMedicine 32, 142–163. doi: 10.1016/j.ebiom.2018.05.027, PMID: 29866590 PMC6021456

[ref35] Rashid M. U. Lao Y. Spicer V. Coombs K. M. (2022). Zika virus infection activates cellular immune responses in Sertoli cells and dysregulates proteins involved in carbohydrate metabolism and cardiovascular disease. Viruses 14:377. doi: 10.3390/v14020377, PMID: 35215967 PMC8878972

[ref36] Shi C. Y. Kingston E. R. Kleaveland B. Lin D. H. Stubna M. W. Bartel D. P. (2020). The ZSWIM8 ubiquitin ligase mediates target-directed microRNA degradation. Science 370:eabc9359. doi: 10.1126/science.abc9359, PMID: 33184237 PMC8356967

[ref37] Sielaff M. Kuharev J. R. Bohn T. Hahlbrock J. Bopp T. Tenzer S.. (2017). Evaluation of FASP, SP3, and iST protocols for proteomic sample preparation in the low microgram range. J. Proteome Res. 16, 4060–4072. doi: 10.1021/acs.jproteome.7b00433, PMID: 28948796

[ref38] Simon P. F. Mccorrister S. Hu P. Z. Chong P. Silaghi A. Westmacott G.. (2015). Highly pathogenic H5N1 and novel H7N9 influenza a viruses induce more profound proteomic host responses than seasonal and pandemic H1N1 strains. J. Proteome Res. 14, 4511–4523. doi: 10.1021/acs.jproteome.5b00196, PMID: 26381135

[ref39] Srivastava M. Zhang Y. Chen J. Sirohi D. Miller A. Zhang Y.. (2020). Chemical proteomics tracks virus entry and uncovers NCAM1 as Zika virus receptor. Nat. Commun. 11:3896. doi: 10.1038/s41467-020-17638-y, PMID: 32753727 PMC7403387

[ref40] Stukalov A. Girault V. Grass V. Karayel O. Bergant V. Urban C.. (2021). Multilevel proteomics reveals host perturbations by SARS-CoV-2 and SARS-CoV. Nature 594, 246–252. doi: 10.1038/s41586-021-03493-4, PMID: 33845483

[ref41] Vanhook A. (2021). Autophagy chokes on SARS-CoV-2. Sci. Signal. 14:abh3628. doi: 10.1126/scisignal.abh3628

[ref42] Vester D. Rapp E. Gade D. Genzel Y. Reichl U. (2009). Quantitative analysis of cellular proteome alterations in human influenza a virus-infected mammalian cell lines. Proteomics 9, 3316–3327. doi: 10.1002/pmic.200800893, PMID: 19504497

[ref43] Wan W. W. Wang L. J. Chen X. Zhu S. L. Shang W. J. Xiao G. F.. (2019). A subcellular quantitative proteomic analysis of herpes simplex virus type 1-infected HEK 293T cells. Molecules 24:4215. doi: 10.3390/molecules24234215, PMID: 31757042 PMC6930547

[ref44] Wang B. Jia X. Yao Q. M. Li Q. He W. W. Li L.. (2019). CEP128 is a crucial risk locus for autoimmune thyroid diseases. Mol. Cell. Endocrinol. 480, 97–106. doi: 10.1016/j.mce.2018.10.017, PMID: 30393005

[ref45] Wu Z. Huang W. Wang X. G. Wang T. Chen Y. D. Chen B.. (2018). Circular RNA CEP128 acts as a sponge of miR-145-5p in promoting the bladder cancer progression via regulating SOX11. Mol. Med. 24:40. doi: 10.1186/s10020-018-0039-0, PMID: 30134837 PMC6069875

[ref46] Wu T. Y. Leng Q. Tian L. Q. (2021). The microRNA-210/Casp8ap2 axis alleviates hypoxia-induced myocardial injury by regulating apoptosis and autophagy. Cytogenet. Genome Res. 161, 132–142. doi: 10.1159/000512254, PMID: 33882492

[ref47] Xin Q. L. Deng C. L. Chen X. Wang J. Wang S. B. Wang W.. (2017). Quantitative proteomic analysis of mosquito C6/36 cells reveals host proteins involved in Zika virus infection. J. Virol. 91, e00554–e00517. doi: 10.1128/JVI.00554-17 28404849 PMC5446628

[ref48] Yu C. Zhang F. C. (2020). LncRNA AC009022.1 enhances colorectal cancer cells proliferation, migration, and invasion by promoting ACTR3B expression via suppressing miR-497-5p. J. Cell. Biochem. 121, 1934–1944. doi: 10.1002/jcb.29428, PMID: 31637768

[ref49] Zhang Y. J. Shen Y. Z. Yin L. Qi T. K. Jia X. F. Lu H. Z.. (2019). Plasma membrane proteomic profile discovers macrophage-capping protein related to latent HIV-1. Curr. HIV Res. 17, 42–52. doi: 10.2174/1570162X17666190506155222, PMID: 31057110

[ref50] Zhao S. S. Geybels M. S. Leonardson A. Rubicz R. Kolb S. Yan Q. X.. (2017). Epigenome-wide tumor DNA methylation profiling identifies novel prognostic biomarkers of metastatic-lethal progression in men diagnosed with clinically localized prostate cancer. Clin. Cancer Res. 23, 311–319. doi: 10.1158/1078-0432.CCR-16-0549, PMID: 27358489 PMC5199634

